# The impact of psychological distress on problematic smartphone use among college students: The mediating role of metacognitions about smartphone use

**DOI:** 10.3389/fpsyg.2022.932838

**Published:** 2022-09-20

**Authors:** Huohong Chen, Jing Ma, Jinliang Guan, Lin Yin, Zifu Shi, Yihan Zhang

**Affiliations:** ^1^School of Educational Science, Hunan Normal University, Changsha, China; ^2^Cognition and Human Behavior Key Laboratory of Hunan Province, Changsha, China; ^3^Key Laboratory of Sports Intelligence Research, Hunan Normal University, Changsha, China; ^4^School of Physical Education, Hunan Normal University, Changsha, China

**Keywords:** depression, anxiety, metacognitions about smartphone use, problematic smartphone use, college students

## Abstract

A mediation model was constructed to clarify the relationship and mechanisms linking psychological distress to problematic smartphone use (PSU), focusing on the mediating role of metacognitions about smartphone use. A questionnaire method was used to investigate psychological distress, metacognitions about smartphone use, and problematic smartphone use among 664 college students. The results showed that (1) psychological distress had a significant positive predictive effect on problematic smartphone use, and (2) there were differences in the underlying mechanisms linking different types of psychological distress to problematic smartphone use. Specifically, negative metacognition about smartphone use partially mediated the relationship between depression and problematic smartphone use, whereas anxiety could act on problematic smartphone use through the parallel mediation of positive metacognition about smartphone use and negative metacognition about smartphone use, with the latter having a greater positive mediating effect than the former. These findings reveal the mechanism of action linking different types of psychological distress to problematic smartphone use from the perspective of the cognitive-behavioral model of pathological Internet use, which has implications for the prevention and intervention of problematic smartphone use among college students.

## Introduction

Problematic smartphone use (PSU) is a non-substance addiction or behavioral addiction that leads to impaired physical, psychological, and social functioning (Liu et al., 2017). Studies have found that individuals with problematic smartphone use have reduced learning engagement (Gao et al., 2021) and reduced sleep quality (Demirci et al., 2015). Although interventions for problematic smartphone use have been developed by scholars from different perspectives (e.g., exercise therapy and environmental interventions), they still lack a high degree of operability (Zhang et al., 2019). Moreover, college students growing up in the digital age are the main users of the smartphone, and thus problematic smartphone use is more common among this group (Qing et al., 2017). Studies have shown that 33.4% of college students have problematic smartphone use (Li et al., 2016), and 58.33% of college students are on the verge of problematic smartphone use (Chen et al., 2016). Therefore, further exploration of the influencing factors and mechanisms of problematic smartphone use among college students could provide new guidelines for the prevention and intervention of problematic smartphone use.

Psychological distress is an adverse emotional experience that occurs when individuals are unable to cope with stress on their own (Holland et al., 2007). Depression and anxiety are the most clinically focused forms of psychological distress (Lovibond and Lovibond, 1995), and prior research has found that individuals in a state of depression and anxiety often cope with their negative experiences through the use of Internet (Li et al., 2019). As the most prevalent Internet access device, smartphone use is the most likely way for individuals with psychological distress to regulate their negative experiences, even though this often does not solve the substantive problem, but rather increases the risk of problematic smartphone use (Yu et al., 2021). Yang et al. (2020) conducted a cross-sectional study with college students as subjects that confirmed this view. In addition, a 6-month follow-up study conducted by Zhou et al. (2021) using high school students as subjects also found that individuals with high depression levels were more likely to be immersed in electronic devices, such as smartphones as a way to avoid stressful events in their lives. More recently, a 10-month longitudinal study by Yu et al. (2021) demonstrated that adolescents with high levels of anxiety were more likely to use their smartphones for social support and thus had a higher likelihood of problematic smartphone use.

However, inconsistent results have also been obtained in some studies. For example, Lee et al. (2020) followed 56 problematic smartphone users and found that initial levels of depression and anxiety did not significantly predict the development of problematic smartphone use episodes 6 months later. This may be related to the statistical method used in the study, i.e., the variables were not statistically analyzed as continuous variables, thus reducing the sensitivity to subtle differences (Lee et al., 2020). Thus, forms of psychological distress, such as depression and anxiety may still be important predictors of problematic smartphone use.

It is far from sufficient to explore the correlations between variables alone. To make more direct recommendations for intervention efforts, one must further examine how psychological distress affects problematic smartphone use (i.e., the mediating mechanisms). The cognitive-behavioral model of pathological Internet use (Davis, 2001) posits that the effects of psychological distress (e.g., depression and anxiety) on individual behavior often work through cognitive factors. An individual’s cognition is a sufficient condition for the emergence of addictive behaviors and is a proximal factor in addictive behaviors. Metacognition, i.e., an individual’s perception of their own cognitive processes and internal states (Wells, 1995), is an alternate perspective for understanding the role of cognition in problematic smartphone use (Shi et al., 2021). Metacognitions about smartphone use have been found to be more important predictors of problematic smartphone use than general metacognition (Shi et al., 2021; Zhou et al., 2021). Therefore, the present study will focus on the mechanisms by which metacognition about smartphone use mediates the relationship between psychological distress and problematic smartphone use among college students.

First, metacognitions about smartphone use can be affected by psychological distress. Metacognitions about smartphone use are the metacognitive beliefs that individuals have about their smartphone use behavior. It includes both positive metacognition (i.e., individuals’ metacognitive beliefs about the emotional and cognitive regulation and social enhancement effects of smartphone use, such as, “Using my smartphone makes me feel happy”) and negative metacognition (i.e., individuals’ metacognitive beliefs about the uncontrollable and harmful effects of smartphone use, such as, “Using my smartphone controls my life”) (Shi et al., 2021). On the one hand, psychological distress may facilitate the activation of positive metacognition about smartphone use. Psychological distress as a negative experience can cause individuals to exhibit symptoms, such as sadness, agitation, and social avoidance (Yang and Zhang, 2021). Individuals tend to seek high-arousal solutions as a way to relieve the discomfort caused by negative experiences (Bryant and Zillmann, 1984). As smartphones are the most convenient and accessible communication tools nowadays, their versatile uses (e.g., socializing, entertainment, and shopping) enable individuals to gain pleasure from them to temporarily alleviate negative experiences (Yang et al., 2020), so college students will be more likely to experience intrusive thinking about the negative experience regulating effects of smartphone use (e.g., “Using a smartphone can reduce my negative feelings”)—in other words, positive metacognition about smartphone use. On the other hand, psychological distress may also promote the activation of individuals’ negative metacognition about smartphone use. When college students with high psychological distress levels repeatedly use smartphones as a way to regulate their negative experiences, their ability to control smartphone use will gradually decrease. This will prevent them from realizing the imbalance in their behaviors and adjusting their internal needs in time so that they are unable to make behavioral adjustments (He et al., 2012). Therefore, over time, they will gradually perceive the uncontrollability of their smartphone use and its negative effects (e.g., “Smartphone use has affected my daily life”)—in other words, negative metacognition about smartphone use.

Second, metacognitions about smartphone use can have an impact on problematic smartphone use. Positive metacognition was expected to play a central role in the pre-engagement of addictive behavior, with negative metacognition activated in the engagement and post-engagement phase, influencing the continuation of addictive behavior (Spada et al., 2013). Although their effects on addictive behaviors vary depending on the stage of behavior (Spada and Caselli, 2017), both the positive and negative metacognitions about smartphone use have been found to be significant predictors of problematic smartphone use (Casale et al., 2020; Shi et al., 2021). In summary, both positive and negative metacognition about smartphone use may be mediating variables in the relationship between psychological distress and problematic smartphone use among college students.

A recent study investigating 535 smartphone users aged 18–65 years found that the following variables all mediated the relationship between psychological distress and problematic smartphone use: positive metacognition about smartphone use having a prosocial role, negative metacognition about smartphone use, positive expectancy about smartphone use, and negative expectancy about smartphone use. However, positive metacognition about smartphone use, which has emotional and cognitive regulation effects, did not mediate the relationship between the two (Casale et al., 2021). Furthermore, this may be related to the overlap of content between the subscale of positive metacognition about smartphone use, which has emotional and cognitive regulation effects, and the subscale of positive expectancy about smartphone use (e.g., “I experience pleasure using my smartphone” vs. “Using my smartphone makes me feel happy”). Positive expectancy about smartphone use refers to individuals’ judgments about the possible positive outcomes of smartphone use (Elhai et al., 2020), whereas positive metacognition about smartphone use is a metacognitive belief about the positive effects of smartphone use (Shi et al., 2021). There is a substantial difference between the two, with the former being a form of cognition and the latter being a form of metacognition (Casale et al., 2021). Although prior research has found that both positive metacognition about smartphone use and positive expectancy about smartphone use positively predict problematic smartphone use (Elhai et al., 2020; Shi et al., 2021), positive metacognition about smartphone use is more focused on motivating individuals to produce smartphone use behavior in the form of thought control (Spada et al., 2015a) and thus should have a more important impact on problematic smartphone use (Casale et al., 2021). However, if two are examined simultaneously, the overlap in the content of the two subscales may make it difficult to truly distinguish the variables at play. Therefore, the present study will separately examine the possible mediating role of metacognitions about smartphone use in the relationship between psychological distress and problematic smartphone use.

In addition, this study will construct a mediational model based on the cognitive-behavioral model of pathological Internet use to explore how psychological distress influences Chinese college students’ problematic smartphone use behavior through metacognitions about smartphone use. The specific research hypotheses are: (1) psychological distress positively predicts problematic smartphone use and (2) metacognitions about smartphone use mediate the relationship between psychological distress and problematic smartphone use.

## Materials and methods

### Participants

A convenience sampling method was used to recruit students (freshmen to seniors) from three universities in Anhui, Hunan, and Sichuan provinces. All students who have taken a mental health education course and had to have used a smartphone. The research protocol was approved by the Ethics Committee of Hunan Normal University in China on 9 October 2021. Informed consent from students was obtained before collecting data. A psychology student who had undergone prior rigorous training as the main test administrator. All students were asked to complete the questionnaires during breaks and were guaranteed strict confidentiality in their answers to the questionnaire. Completing the questionnaire took approximately 15 min. Data were collected by filling out questionnaires on the spot.

Overall, a total of 724 questionnaires were collected. A total of 664 valid questionnaires (*F* = 49.50%, mean age = 19.25 ± 1.17 years) were obtained after eliminating invalid questionnaires containing missing data and extreme values. Among them, 2.70% of participants used smartphones for ≤ 2 h, 24.40% for 2–4 h, 38.60% for 4–6 h, 23.20% for 6–8 h, and 11.10% for ≥ 8 h per day.

### Measures

#### Psychological distress

The depression subscale and anxiety subscale of the Depression Anxiety Stress Scales developed by Lovibond and Lovibond (1995) and revised by Wang et al. (2016) were used. Both subscales include 7 items, and sample items are “I feel thirsty” and “I cannot feel pleasant or comfortable anymore,” Each item of the questionnaire is scored from 0 to 3, and so total scores range from 0 to 21 for each of the depression and anxiety subscales. The higher the total score on each subscale, the more severe the degree of psychological distress. The subscales have shown good psychometric properties as a measure to assess depression and anxiety in Chinese college students (Wang et al., 2016). In the current study, Cronbach’s alpha (α) = 0.86 for the depression subscale and α = 0.82 for the anxiety subscale.

#### Metacognitions about smartphone use

This self-reported measurement was developed by Casale et al. (2020) and revised by Shi et al. (2021). It comprises 24 items, including two dimensions: positive metacognition about smartphone use (14 items) and negative metacognition about smartphone use (10 items). Sample items are, “Using my smartphone makes me feel happy” and “Using my smartphone controls my life.” Each item of the questionnaire is scored from 1 to 4, and so the total scores for the positive metacognition about smartphone use subscale and the negative metacognition about smartphone use subscale were 4–56 and 4–40, respectively. Higher scores indicate higher levels of dysfunctional metacognitions associated with smartphone use. This measurement demonstrated effective reliability and validity in a previous study (Shi et al., 2021). In the current study, Cronbach’s alpha (α) = 0.92 for the positive metacognition about smartphone use subscale and α = 0.88 for the negative metacognition about smartphone use subscale.

#### Problematic smartphone use

This self-reported measurement scale was developed by Su et al. (2014). The scale comprises a total of 22 items. Participants gave their answers on a five-point Likert scale from 1 = *do not agree* to 5 = *agree very much*. A sample item is, “I keep an eye on the latest app version and download it to my smartphone.” Higher scores represent higher levels of PSU. This measurement has demonstrated effective reliability and validity among Chinese college students (Su et al., 2014). In the present study, Cronbach’s alpha of the scale was 0.93.

### Data collection and analysis

Data were processed using SPSS 26.0 statistical software. All of the variables in this study were measured using the subjects’ self-reports, which may introduce common method bias. To reduce this possibility, common method bias was reduced and examined through procedural control and statistical control with reference to previous studies (Zhou and Long, 2004). For procedural control, the following were used to design the questionnaire: (a) subjects completed the questionnaire anonymously; and (b) some entries were scored using reverse scoring. Statistically, the Harman one-way test was used to test for common method bias. An unrotated principal component factor analysis of all items revealed that a total of 10 factors had eigen root values greater than 1, and the first common factor explained only 24.22% (i.e., less than 40%) of the total variance, indicating that there was no serious common method bias in this study (Podsakoff et al., 2003).

## Results

### Descriptive statistics and correlation analysis

In the first step, we drew a comparison between male and female participants in relation to the variables of the study. As shown in Table 1, we found that the female participants scored higher on problematic smartphone use. Then correlation analysis results (in Table 2) showed that depression and anxiety, negative metacognition about smartphone use, and problematic smartphone use were significantly and positively correlated, but depression was not significantly correlated with positive metacognition about smartphone use. Anxiety was significantly and positively correlated with positive metacognition about smartphone use, negative metacognition about smartphone use, and problematic smartphone use. Positive metacognition about smartphone use was significantly and positively correlated with negative metacognition about smartphone use and problematic smartphone use. Negative metacognition about smartphone use and problematic smartphone use was significantly and positively correlated.

**TABLE 1:** Differences between male and female across variables.

	Male		Female		
	*M*	*SD*	*M*	*SD*	*t*
1. Depression	1.95	3.12	1.61	2.64	1.53
2. Anxiety	2.78	3.33	2.67	3.10	0.48
3. PM	32.92	8.54	32.73	7.52	0.31
4. NM	17.92	6.29	18.35	5.89	−0.91
5. PSU	53.59	16.92	57.06	15.72	−2.73**

PM, positive metacognition about smartphone use; NM, negative metacognition about smartphone use; PSU, problematic smartphone use.

**p < 0.01.

**TABLE 2:** Correlations between the study variables.

	1	2	3	4	5
1. Depression	−				
2. Anxiety	0.79**	−			
3. PM	0.07	0.09*	−		
4. NM	0.36**	0.33**	0.29**	−	
5. PSU	0.30**	0.38**	0.30**	0.62**	−

PM, positive metacognition about smartphone use; NM, negative metacognition about smartphone use; PSU, problematic smartphone use.

*p < 0.05, **p < 0.01.

### Mediated model test

First, all predictor variables in this study had variance inflation factors of no higher than 2.81, indicating that there was no problem of multicollinearity. The mediating effect of metacognitions about smartphone use between psychological distress and problematic smartphone use was then analyzed using Model 4 in the SPSS macro program PROCESS, developed by Hayes (2013). The bias-corrected non-parametric percentile Bootstrap method is a test with high precision and testing power (Fang and Zhang, 2012), so it was also used to test the validity of the mediation model. The Bootstrap sampling size was set to 5,000 times and the confidence interval had a confidence level of 95%. Moreover, according to prior studies, there are significant gender and age differences in problematic smartphone use among college students (Wu et al., 2019; Shi et al., 2021), so this study used both as control variables in the mediation analysis.

**FIGURE 1:**
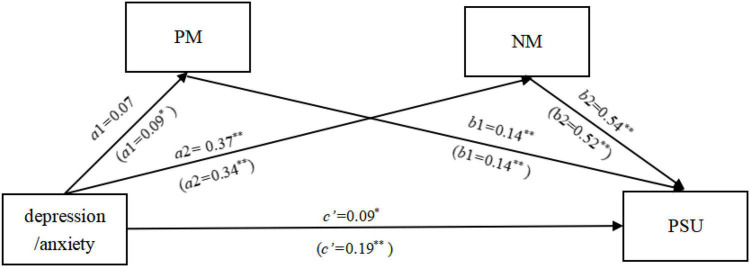
Results of the mediated model among the key study variables. The values in parentheses are the values calculated when anxiety is the independent variable; PM, positive metacognition about smartphone use; NM, negative metacognition about smartphone use; PSU, problematic smartphone use; *p < 0.05, **p < 0.01.

The results of the study (as shown in Figure 1) showed that, with gender and age controlled for, depression was not a significant predictor of positive metacognition about smartphone use (β = 0.07, *p* > 0.05), but was a significant positive predictor of negative metacognition about smartphone use (β = 0.37, *p* < 0.01). With depression, positive metacognition about smartphone use, and negative metacognition about smartphone use entered into the regression equation simultaneously, they all significantly and positively predicted problematic smartphone use (β = 0.09, *p* < 0.05; β = 0.14, *p* < 0.01; and β = 0.54, *p* < 0.01). Furthermore, as shown in Table 3, the Bootstrap 95% confidence interval for the pathway of positive metacognition about smartphone use contained 0, whereas the Bootstrap 95% confidence interval for the pathway of negative metacognition about smartphone use did not contain 0. This indicates that only negative metacognition about smartphone use partially mediated the relationship between depression and problematic smartphone use.

**TABLE 3:** The mediation effect test.

Pathway	Effect	BootSE	BootLLCI	BootULCI
Depression → PM → PSU	0.010	0.005	0	0.023
Depression → NM → PSU	0.199	0.024	0.153	0.247
PM – NM	−0.188	0.023	−0.234	−0.143
Anxiety → PM → PSU	0.013	0.006	0.003	0.027
Anxiety → NM → PSU	0.173	0.021	0.132	0.216
PM – NM	−0.160	0.021	−0.202	−0.119

PM, positive metacognition about smartphone use; NM, negative metacognition about smartphone use; PSU, problematic smartphone use; PM-NM, the mediating effect values of the two are subtracted.

In addition, the findings revealed (as shown in Figure 1) that, controlling for gender and age, the positive predictive effects of anxiety on both positive metacognition about smartphone use and negative metacognition about smartphone use were significant (β = 0.09, *p* < 0.05; and β = 0.34, *p* < 0.01), and the positive predictive effects of both positive metacognition about smartphone use and negative metacognition about smartphone use on problematic smartphone use was also significant (β = 0.14, *p* < 0.01; and β = 0.52, *p* < 0.01). In addition, the direct predictive effect of anxiety on problematic smartphone use was significant (β = 0.19, *p* < 0.01). Moreover, the Bootstrap 95% confidence intervals for pathways of both positive metacognition about smartphone use and negative metacognition about smartphone use did not contain 0 (as shown in Table 3). This suggests that anxiety not only can directly influence problematic smartphone use but also can act on problematic smartphone use through the parallel mediation of both positive metacognition about smartphone use and negative metacognition about smartphone use. In particular, the magnitude of the mediating effect of negative metacognition about smartphone use was greater than that of positive metacognition about smartphone use.

## Discussion

Based on the cognitive-behavioral model of pathological Internet use (Davis, 2001), this study examined the relationship between psychological distress and problematic smartphone use among college students, as well as the mediating role of metacognitions about smartphone use between the two. The results indicate that depression and anxiety can directly predict problematic smartphone use among college students and can also have indirect effects on problematic smartphone use through different mediating pathways. These findings further elucidate the mechanism of action by which psychological distress affects problematic smartphone use, which has implications for the prevention and intervention of problematic smartphone use among college students.

Consistent with the results of prior studies, this study found that psychological distress significantly and positively predicts problematic smartphone use among college students. On the one hand, self-determination theory suggests that the satisfaction of individuals’ relationship needs, autonomy needs, and competence needs to influence the development of their adaptive behavior (Deci and Ryan, 2000). Psychological distress as a negative experience can leave college students with unmet psychological needs, such as relationships, autonomy, and competence (Wang et al., 2020). If real-life needs are not well met, individuals will find other ways to satisfy these needs, such as using the Internet (Liu et al., 2016). Previous research has found that people with lower satisfaction with psychological needs in real life are more likely to seek social connections on the Internet (Wang et al., 2015). Nowadays, smartphones, as the largest Internet-using terminal (CNNIC, 2019), can satisfy psychological needs that people cannot be satisfied in society, which may be one of the reasons for problematic smartphone use among college students (Cui et al., 2014). On the other hand, psychological distress can weaken college students’ self-control and reduce their resistance to addictive objects by inhibiting the activity of executive functions (Mitchell and Phillips, 2007). The process of coping with psychological distress is essentially a process of ego depletion (Muraven and Baumeister, 2000). When self-control resources are depleted, the individual’s executive control function decreases, making it difficult to maintain normal cognitive activities, such as attention allocation and risk assessment in subsequent decision-making, which in turn can easily trigger impulsive behaviors (Dou et al., 2014), such as problematic smartphone use (Tong et al., 2019). This suggests that how to reduce the level of psychological distress among college students is an important aspect of the prevention and intervention of problematic smartphone use in the future.

Furthermore, in contrast to the results of Casale et al. (2021), the mechanisms underlying the effects of different types of psychological distress on problematic smartphone use differed. Specifically, depression has an indirect effect on problematic smartphone use primarily through negative metacognition about smartphone use, whereas anxiety can act on problematic smartphone use through a parallel mediation of both positive and negative metacognitions about smartphone use. This is an important finding that seems to suggest that anxious individuals are more likely to use smartphones as a form of self-regulation, whereas depressed individuals may use smartphones to satisfy other psychological needs, such as the acquisition of a sense of control (Cheng et al., 2013). However, these may weaken the individual’s control over smartphone use, and the weakened behavioral inhibition may prevent the individual from noticing the imbalance in smartphone use and adjusting their internal needs in time (He et al., 2012), activating negative metacognition about smartphone use. The perceived failure of self-regulation and the harmful effects of imbalanced smartphone use reinforce negative repetitive thoughts and negative experiences (Casale et al., 2021), which further compel individuals to continue using the smartphone to regulate their imbalanced internal state (Spada et al., 2015b), ultimately leading to addiction (Casale et al., 2020; Shi et al., 2021). Therefore, in future practice, educators and psychologists can reduce the likelihood of problematic smartphone use by reducing the activation of metacognitions about smartphone use in college students through techniques, such as detached mindfulness and situational attention refocusing (Wells, 2000).

In addition, this study found that negative metacognition about smartphone use positively mediates the relationship between anxiety and problematic smartphone use more than positive metacognition about smartphone use. This finding reaffirms that negative metacognition about smartphone use is a more important predictor of problematic smartphone use than positive metacognition about smartphone use (Casale et al., 2020; Shi et al., 2021). However, it is worth noting that the subjects selected for this study were not distinguished between normal and clinical individuals. Instead, Caselli et al. (2018), who distinguished subjects between normal gamblers and pathological gamblers, found that positive metacognition was more effective in predicting the long-term severity of addiction in normal gambler subjects compared with negative metacognition, but the opposite result was obtained in pathological gambler subjects. Therefore, future research could further examine group differences in the effects of positive and negative metacognition about smartphone use on problematic smartphone use.

The present study has some limitations that could be rectified in future studies. First, this study used a cross-sectional research design, and thus the findings could not reveal causal relationships. Second, this study only explored the effects of depression and anxiety, two of the more common types of psychological distress, on problematic smartphone use. Stress, which is also a type of psychological distress and an intrinsic form of depression and anxiety (Lovibond and Lovibond, 1995), will need to be examined in future studies.

## Conclusion

(1) Psychological distress significantly and positively predicts problematic smartphone use among college students.

(2) The mechanisms of action between different types of psychological distress and problematic smartphone use are different. Specifically, negative metacognition about smartphone use partially mediates the relationship between depression and problematic smartphone use, whereas anxiety can act on problematic smartphone use through the parallel mediation of positive metacognition about smartphone use and negative metacognition about smartphone use, with the latter having a greater positive mediating effect than former.

## Data availability statement

The original contributions presented in this study are included in the article/supplementary material, further inquiries can be directed to the corresponding authors.

## Ethics statement

The studies involving human participants were reviewed and approved by Ethics Committee of Hunan Normal University. The patients/participants provided their written informed consent to participate in this study.

## Author contributions

HC and ZS designed the study and wrote the protocol. JG, LY, and JM conducted literature searches and provided summaries of previous research studies. YZ and JM conducted the statistical analysis. HC wrote the first draft of the manuscript. All authors approved the final manuscript.
